# Bone plate repositioned over the antrostomy after sinus floor elevation: an experimental study in sheep

**DOI:** 10.1186/s40729-020-0207-1

**Published:** 2020-03-18

**Authors:** Alessandro Perini, Giada Ferrante, Stefano Sivolella, Joaquín Urbizo Velez, Franco Bengazi, Daniele Botticelli

**Affiliations:** 1 Department of Neuroscience, Division of Dentistry, University of Padua, Via Giustiniani 2, 35128 Padua, Italy; 2 Faculty of Dentistry, University of Medical Science, Havana, Cuba; 3 ARDEC Academy, Ariminum Odontologica, Rimini, Italy

**Keywords:** Animal study, Sinus floor elevation, Bone window, Bone, Bone plate, Polylactic membrane, Window repositioning, Bone graft

## Abstract

The objective of this study was to compare the healing of the augmented sinus at which the antrostomy was covered with a membrane or the repositioned bone plate.

Eight sheep underwent bilateral maxillary sinus floor augmentation. The control site was covered with a resorbable membrane, while at the experimental site the bone plate was repositioned, and both were secured with cyanoacrylate. Animals were euthanised after 4 months and histomorphometric analysis was performed.

A large amount of the graft appeared to be partially interpenetrated by the newly formed bone. Statistical analysis demonstrated different percentages of the new bone and bone interpenetrated to the graft between test and control site in the close-to-window area respectively 22.1 ± 12.6 vs 7.5 ± 4.5 (*P* = 0.028) and 66.1 ± 14.7 vs 44.2 ± 15.1 (*P* = 0.046). Other areas showed no difference in the bone and graft amount. More bone was found at the edges of the antrostomy in the experimental site, without statistical significance. In the centre of the antrostomy, the replaced bony window appeared bonded to the newly formed bone. No remnants and no biological response to cyanoacrylate were observed.

The repositioning of the bony window after sinus floor elevation in sheep led to a larger amount of newly formed bone in the close-to-window zone of the grafted area. The bony window appeared partially bonded to the new bone. Newly formed bone was found interpenetrating the graft granules.

## Introduction

Sinus floor elevation is a commonly used technique to increase bone volume in the posterior maxilla prior to implant placement. This procedure was first developed by Tatum in 1977 [1], modified by Boyne and James in 1980 [2], and further modified over time. In this well-described technique, a bony window is created on the lateral wall of the sinus with a round burr, and the membrane elevated. Different materials have been used to fill the created space, such as autologous bone [3–6], bone substitutes alone [7], bone substitutes in combination with autologous bone [8], or no material [9].

Different methods of closing the antrostomy have also been used. The antrostomy can be left open, suturing the mucosa directly over the grafted material, or it can be covered with a resorbable [10] or non-resorbable membrane [11], or closed by repositioning the bony window [9, 12–14].

A systematic review and meta-analysis reported that the use of a resorbable membrane to cover the antrostomy resulted in better outcomes in terms of implant survival rate compared with no covering [10]. However, in another systematic review with meta-analysis, no difference in the bone formation was seen between the two methods [15].

A different method described in the literature is the repositioning of the removed bony plate onto the antrostomy. This technique was described by Lundgren et al. in 2004 [9]: a bevelled osteotomy with a thin reciprocating saw was created and the bony plate removed. After the sinus elevation, the bony plate was repositioned over the window.

In an experimental study in rabbits [16], a bilateral sinus augmentation was performed using a resorbable beta-tricalcium phosphate (β-TCP). The antrostomy was covered either with a collagen membrane or with a repositioned bony plate without any fixation. A faster and greater bone formation was reported in the repositioned bone plate sites.

To prevent the movement of the bony plate and to gain adequate stability, cyanoacrylate has been suggested to glue the plate to the bony edges of the antrostomy [17]. Cyanoacrylates (as methyl 2-cyanoacrylate or ethyl 2-cyanoacrylate) are widely used in surgery and have shown good compatibility [18] and biomechanical strength for fixation of grafts [19]. In an experiment in rabbits [17], the antrostomy at the control site was covered with a collagen membrane, while at the test side the bony window was fixated with cyanoacrylate. After 8 weeks of healing, no differences were seen in the bone formation within the sinus. However, the bone plate was incorporated into the antrostomy by the newly formed bone, while at the collagen membrane sites the healing was incomplete.

No data are available in larger animal models or with larger antrostomy, so the need exists for further such evidence on the healing of the repositioned bone plate and augmented sinus. Hence, the aim of the present experiment was to compare the healing of the augmented sinus where the antrostomy was covered with a resorbable membrane or a repositioned bone plate, both fixed with cyanoacrylate.

## Materials and methods

The research protocol was submitted to and approved by the Ethical Committee of the University of Medical Sciences, School of Dentistry, Havana, Cuba (prot. 013/2013).

### Sample and location for animal treatment and maintenance

Eight female Pelibuey sheep, with a mean body weight of approximately 35 kg and a mean age of approximately 3 years, were provided by the Centro Nacional para la Producción de Animales de Laboratorio (CENPALAB) in Havana, Cuba. The surgical sessions were performed at the Centro de Cirugía Experimental (CENCEX) Facultad de Medicina “Victoria de Girón”, Universidad de Ciencias Médicas in Havana, Cuba. The animals were kept at the facilities of CENPALAB during the experimental period.

### Randomisation and allocation concealment

The ARRIVE checklist was followed as a guide. According to the Three R requirements [20], aiming to reduce the number of animals, the interferences among animals was eliminated, adopting a split-mouth design. No previous experiments were available that studied healing at the repositioned bony plate, so to describe the event of healing with sufficient approximation, a relevant difference in bone formation within the sinus was set to 10%, assuming a standard deviation of approximately 8%. Considering these values clinically relevant, seven pairs of subjects were calculated to be able to reject the null hypothesis that this response difference is zero with a power of 0.8 and *α* = 0.05.

Two different procedures for antrostomy closure were adopted, one using a resorbable membrane (control site) and the other repositioning the bone plate (experimental site). The randomisation was performed electronically (www.randomization.com) by an author not involved in any surgical procedures (DB). The surgeon (AP) was informed of the treatment randomly selected only at the end of the grafting procedure.

The examiner of the histological slides (AP) was carefully trained before the evaluation. The measurements were performed twice, and mean values used.

### Anaesthetic procedures

The animals fasted for 24 h preoperatively, but were allowed to drink water ad libitum. Anaesthesia was induced by 0.4 mg/kg of midazolam (Dormicum; Roche, Basel, Switzerland) and 10 mg/kg of ketamine (Ketamina-50; Liorad, Havana, Cuba), and orotracheal intubation was performed. The anaesthesia was maintained with a mixture of oxygen and 2–3% isoflurane (Isoflurane-vet; Merial, Toulouse, France) at a rate of 5 L/min. The surgical sites were rinsed with 0.12% chlorhexidine digluconate (Periogard™; Colgate-Palmolive Ltd, New York, NY, USA) and trichotomy was performed. After general anaesthesia, 1.5–2 cc of 2% mepivacaine HCI with 1:100,000 epinephrine was injected at the surgery site. All surgeries were performed under sterile conditions, using good clinical and laboratory practices.

### Clinical procedures

**Fig. 1:**
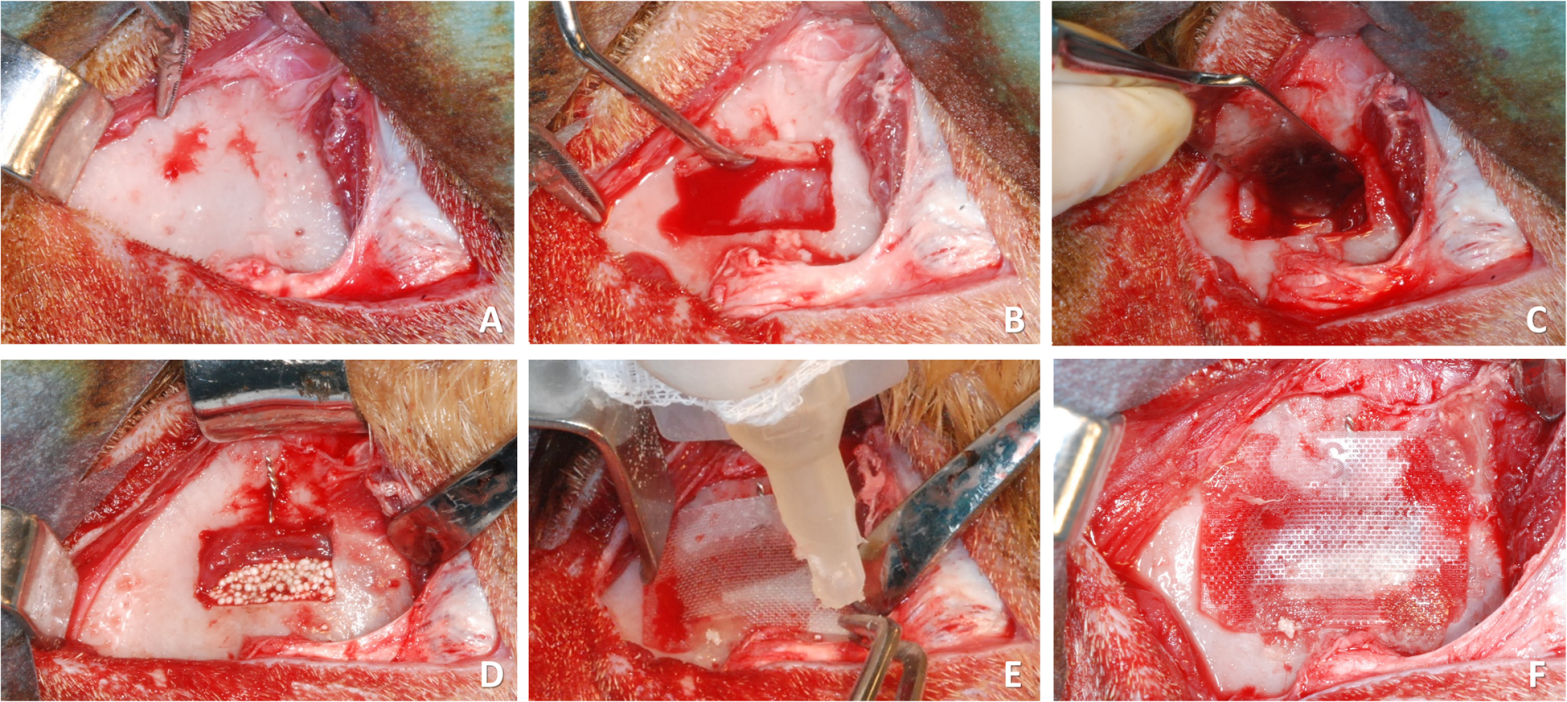
Clinical view at a membrane site. **a** Skin and periosteum were separately elevated, and the facial sinus wall exposed. **b** A 12 × 8-mm window was cut and removed. **c** The Schneiderian membrane was carefully elevated. **d** A twisted wire was inserted in the middle of the long side of the window and the elevated sinus was grafted. **e** At the control site, a resorbable membrane was placed and secured with cyanoacrylate. **f** Membrane in situ

Through an extra-oral approach, an oblique incision was made bilaterally along the sagittal axis between the facial tuberosity and the inferior orbital rim. The skin and periosteum were elevated separately, and the bony facial sinus wall was exposed on both sides of the maxilla (Fig. 1a).

A 12-mm large and 8-mm high antrostomy was prepared using a burr (H254E Komet Dental, Trophagener Weg 25, Lemgo, Germany) and the bone plate was removed bilaterally (Fig. 1b). The sinus mucosa was subsequently carefully elevated with sinus floor elevators, exposing the medial bony plate of the maxillary sinus (Fig. 1c). A landmark was positioned, twisting a steel wire through a hole above the access window (Fig. 1d).

After sinus mucosa elevation, the obtained space was filled with a biphasic calcium phosphate (60% HA, 40% β-TCP) (Easy-graft^TM^ CRYSTAL; Sunstar GUIDOR, Etoy, Switzerland) on both sides (Fig. 1d).

**Fig. 2:**
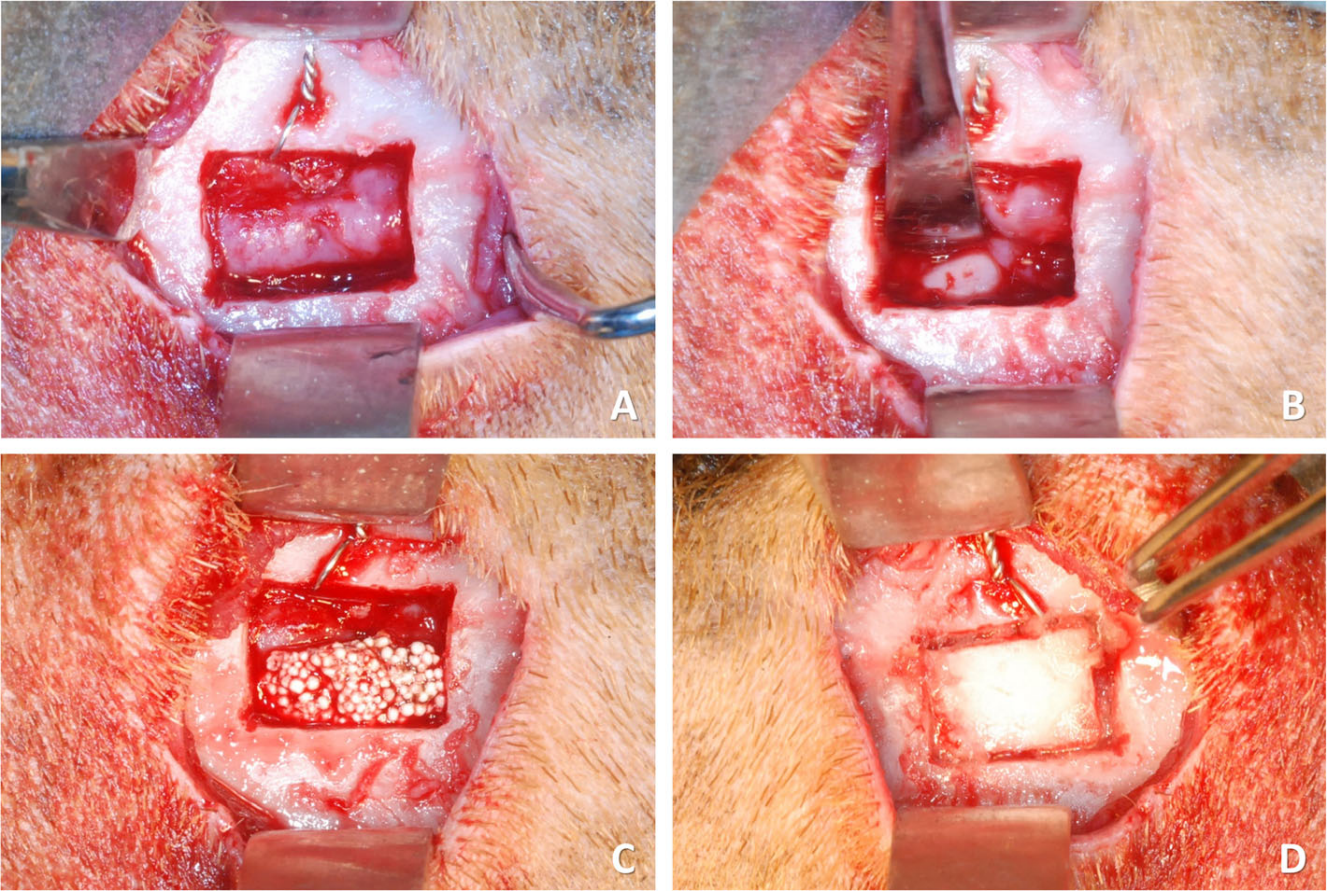
Clinical view at a bone plate site. **a** The bone window was removed. **b** The sinus mucosa was carefully elevated, and a twisted wire was placed. **c** The elevated sinus was grafted. **d** The access bony window was repositioned and secured with cyanoacrylate

Experimental and control sites were randomly chosen. The control site was covered with a polylactic acid blended with a citric acid ester membrane (GUIDOR® matrix barrier; Sunstar Americas Inc., Chicago, IL, USA) and secured with a minimum amount of cyanoacrylate glue (Indermil® x fine; Henkel-Loctite Corp. Dublin, Republic of Ireland) accurately positioned in four points (one at each corner; Fig. 1e, f). Cyanoacrylate glue was stored at 4 °C and used immediately after leaving the refrigerator, to keep the viscosity low. At the experimental site, similar procedures were applied (Fig. 2a–c), and the bony window was repositioned in place and secured with the four points of cyanoacrylate (one at each corner; Fig. 2d). Suturing in layers was then performed.

### Maintenance after surgery

Gentamicin (Gentamicin-5® Bela-Pharm GmbH, Vechta, Germany) 8 mL/100 kg was administered every 12 h during the first postoperative day, and every 24 h during the following 3 days. The sheep were kept in an animal house individually, in a roofed shed to reduce distress after surgery, with a concrete floor. The boxes were cleaned daily and the animals had free access to water. The diet was based on a balanced specific food made of cereals, protein, and concentrates of vitamins and minerals, with added green forages. The wounds were cleaned by the veterinarians of the centre every day during the first week, and then inspected three times per week for clinical signs of complications for the duration of the experiment.

### Euthanasia

After 4 months, the animals were anaesthetised and then euthanised with an overdose of pentobarbital sodium and subsequently perfused with 10% formalin. The maxilla was retrieved en bloc, trimmed, and immersed in formalin solution.

### Histological preparation

All histological procedures were performed in the Laboratorio de Histologıa de la Facultad de Odontologıa de la Universidad de Ciencia Medica in Havana, Cuba. Bilateral maxillary block sections, each containing one sinus, were obtained and maintained in a 4% formaldehyde solution. The blocks were then cut in a buccolingual plane at the level of the landmark reference, using a diamond band saw fitted in a precision slicing machine (Exakt; Apparatebau, Norderstedt, Germany). One hemiblock was dehydrated in a series of graded ethanol and subsequently embedded in resin (Technovit® 7200 VLC; Kulzer, Friedrichsdorf, Germany). One section was obtained and reduced to a thickness of approximately 60 μm using a cutting-grinding device (Exakt®; Apparatebau, Norderstedt, Germany). Subsequently, the histological slides were stained with Stevenel’s blue and alizarin red and examined under a light microscope for histometric analysis.

### Histological evaluations

The histological measurements were performed using an Eclipse Ci microscope (Nikon Corporation, Tokyo, Japan), equipped with a digital video camera (Digital Sight DS-2Mv, Nikon Corporation, Tokyo, Japan), connected to a computer and using the software NIS-Elements D 4.10 (Laboratory Imaging, Nikon Corporation, Tokyo, Japan).

**Fig. 3:**
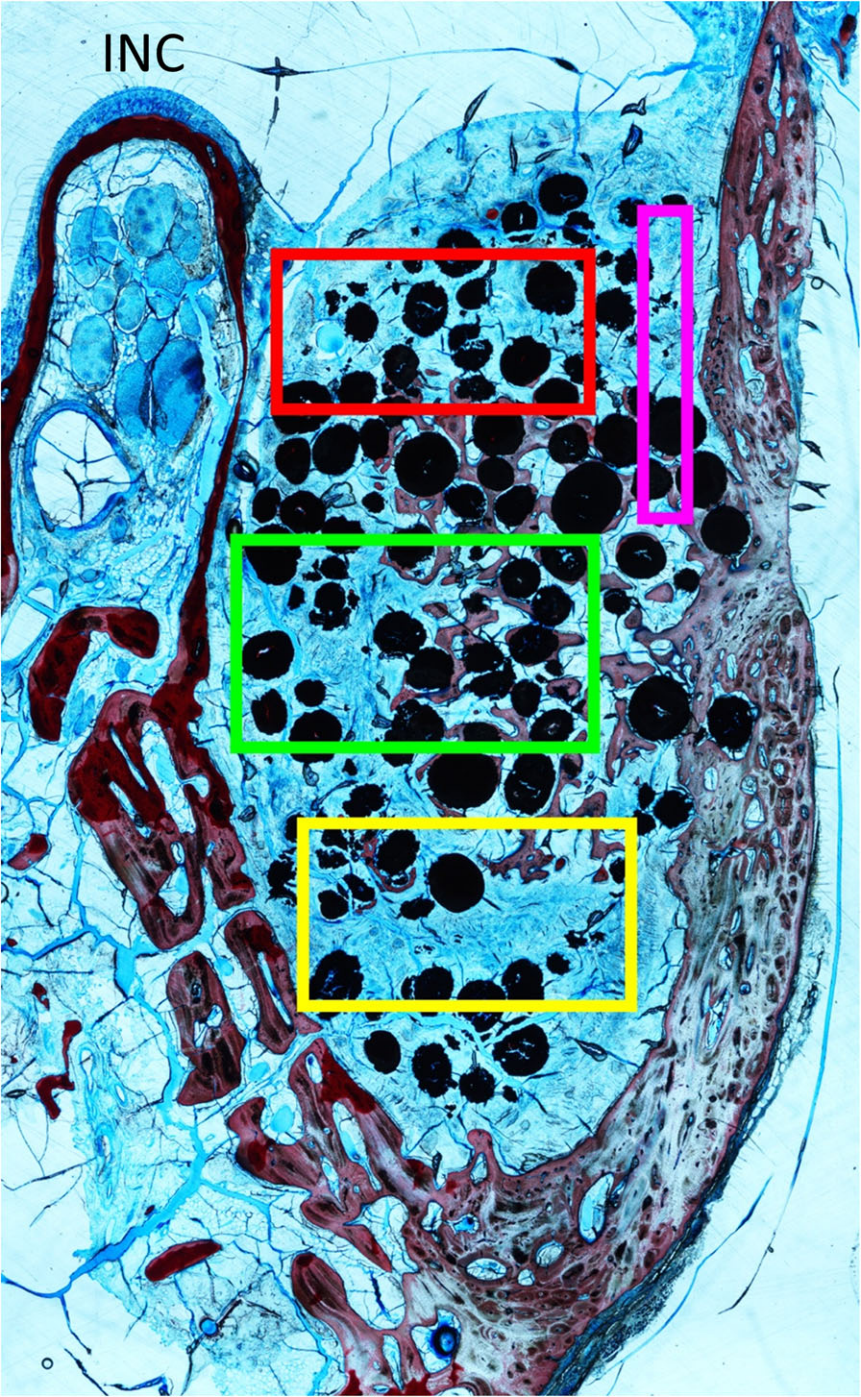
**a** The elevated area was divided into four regions for morphometric analysis. RED: submucosa; GREEN: middle; YELLOW: base; PURPLE: close-to-window. INC: top of the infraorbital nerve canal

Histological measurements within the augmented sinus were performed in four different regions: (i) subjacent to the sinus elevated mucosa (submucosa), (ii) centre of the grafted area (middle), (iii) the original base of the sinus (base), and (iv) immediately under the replaced bone window or the membrane (sub-window) (Fig. 3a).

In the antrostomy area, histology was performed in two regions: (i) the bony window or membrane area (centre) and (ii) the osteotomy regions on the maxilla (edges) (Fig. 3b).

A point counting procedure [21] was performed to identify the tissue composition within the described regions. A lattice, with squares of 75 μm in dimensions, was superposed over the tissues at a magnification of × 100.

The percentages of the new mineralised bone, soft or connective tissue, pure graft, graft interpenetrated by bone, and remnants of cyanoacrylate were evaluated. The total tissue percentages in the elevated space that included submucosa, middle, base, and sub-window regions were also calculated.

### Data analysis

Mean values and standard deviations (SDs) as well as the 25th, 50th (median), and 75th percentiles were calculated for each outcome variable. The main outcome variable was the percentage of the new mineralised bone within the sinus.

The IBM SPSS statistics software (IBM Inc., Chicago, IL, USA) was adopted for analyses. The Wilcoxon test was used to assess the differences between the repositioned bone plate and collagen membrane sites. The level of significance was set at *α* = 0.05.

## Results

During surgery, one sheep showed acute sinusitis at the test site. The sinus mucosa was perforated to allow sinus drainage and surgery was completed. During the healing period, no evident clinical complications were observed.

At the histomorphometric analysis, one sinus of the control group and one of the test group (corresponding to the sinusitis case) appeared to have lost almost all biomaterial. The results have thus been calculated on *n* = 6 and can be seen in Table 1. In one test site, the repositioned bone plate was apparently lost during the histological processing. Mean values and SDs are indicated in text and tables. The statistically significant differences are marked with an asterisk (*).

**Table 1:** Percentages of the various tissues within the elevated area after 4 months of healing. Mean values ± standard deviations (*P* values) and median (25%; 75% percentiles)

		New bone	Soft tissue	Pure graft	Interpenetrated graft	Composite bone
**Replaced window**	**Total**	16.4 ± 5.6 18.8 (13.8; 20.3)	32.9 ± 8.0 31.2 (27.7; 37.0)	13.6 ± 4.2 12.0 (10.8; 16.2)	37.1 ± 7.5 34.4 (31.7; 43.4)	53.5 ± 7.6 52.4 (50.8; 57.8)
	**Base**	15.0 ± 7.2 16.4 (10.1; 20.1)	38.6 ± 14.3 34.5 (28.9; 47.1)	13.4 ± 6.4 15.3 (13.1; 17.0)	33.1 ± 11.1 33.1 (26.8; 40.0)	48.0 ± 18.1 49.9 (36.9; 59.7)
	**Middle**	16.9 ± 7.3 18.1 (11.2; 22.8)	29.7 ± 4.9* 29.8 (26.6; 32.4)	14.2 ± 5.1 14.2 (10.2; 17.6)	39.2 ± 7.6 37.2 (34.3; 39.1)	56.1 ± 7.0 57.2 (51.1; 61.3)
	**Submucosa**	11.5 ± 7.6 10.6 (7.7; 13.3)	41.7 ± 18.5 34.0 (31.3; 43.6)	14.6 ± 5.9 12.7 (10.0; 18.0)	32.3 ± 16.9 31.3 (25.5; 46.0)	43.8 ± 19.6 46.8 (40.9; 56.6)
	**Close-to-window**	22.1 ± 12.6* 19.6 (14.5; 28.1)	21.8 ± 10.5* 21.8 (15.6; 26.2)	12.1 ± 10.1 10.6 (6.9; 15.1)	43.9 ± 20.5 40.2 (29.2; 52.0)	66.1 ± 14.7* 64.3 (57.1; 69.5)
**Membrane**	**Total**	12.0 ± 3.7 (*P* = 0.249) 12.9 (12.3; 13.4)	37.4 ± 9.3 (*P* = 0.173) 35.2 (30.7; 39.8)	15.3 ± 3.9 (*P* = 0.249) 14.0 (12.8; 18.3)	35.3 ± 6.8 (*P* = 0.345) 34.8 (30.4; 41.1)	47.3 ± 9.9 (*P* = 0.249) 47.3 (43.2; 54.3)
	**Base**	14.0 ± 7.5 (*P* = 0.463) 11.6 (8.8; 20.7)	39.9 ± 12.5 (*P* = 0.753) 36.6 (31.0; 47.5)	16.4 ± 6.9 (*P* = 0.686) 13.2 (12.2; 19.7)	29.6 ± 11.4 (*P* = 0.249) 34.3 (22.1; 37.6)	43.6 ± 16.9 (*P* = 0.917) 46.5 (30.1; 56.7)
	**Middle**	14.5 ± 4.0 (*P* = 0.674) 14.4 (13.1; 17.3)	37.5 ± 10.1* (*P* = 0.046) 37.2 (28.3; 45.0)	13.5 ± 7.5 (*P* = 0.686) 15.1 (7.1; 17.9)	34.5 ± 11.5 (*P* = 0.249) 32.6 (26.0; 39.3)	49.0 ± 12.0 (*P* = 0.075) 45.7 (39.9; 55.9)
	**Submucosa**	11.9 ± 5.6 (*P* = 0.917) 14.1 (12.7; 14.5)	34.7 ± 11.6 (*P* = 0.753) 30.1 (27.3; 39.2)	13.0 ± 10.3 (0.753) 9.5 (6.0; 17.8)	40.4 ± 14.7 (0.463) 42.7 (38.2; 48.7)	52.3 ± 15.9 (0.528) 56.8 (42.5; 62.5)
	**Close-to-window**	7.5 ± 4.5* (*P* = 0.028) 8.8 (4.2; 9.6)	37.5 ± 11.5* (*P* = 0.046) 33.8 (32.1; 34.4)	18.3 ± 9.4 (*P* = 0.249) 18.0 (14.9; 18.4)	36.7 ± 11.5 (*P* = 0.249) 38.0 (30.6; 42.1)	44.2 ± 15.1* (*P* = 0.046) 49.5 (35.3; 51.4)

*P < 0.05

### Histological analysis in the elevated area (Table 1, Figs. 4, 5, and 6)

**Fig. 4:**
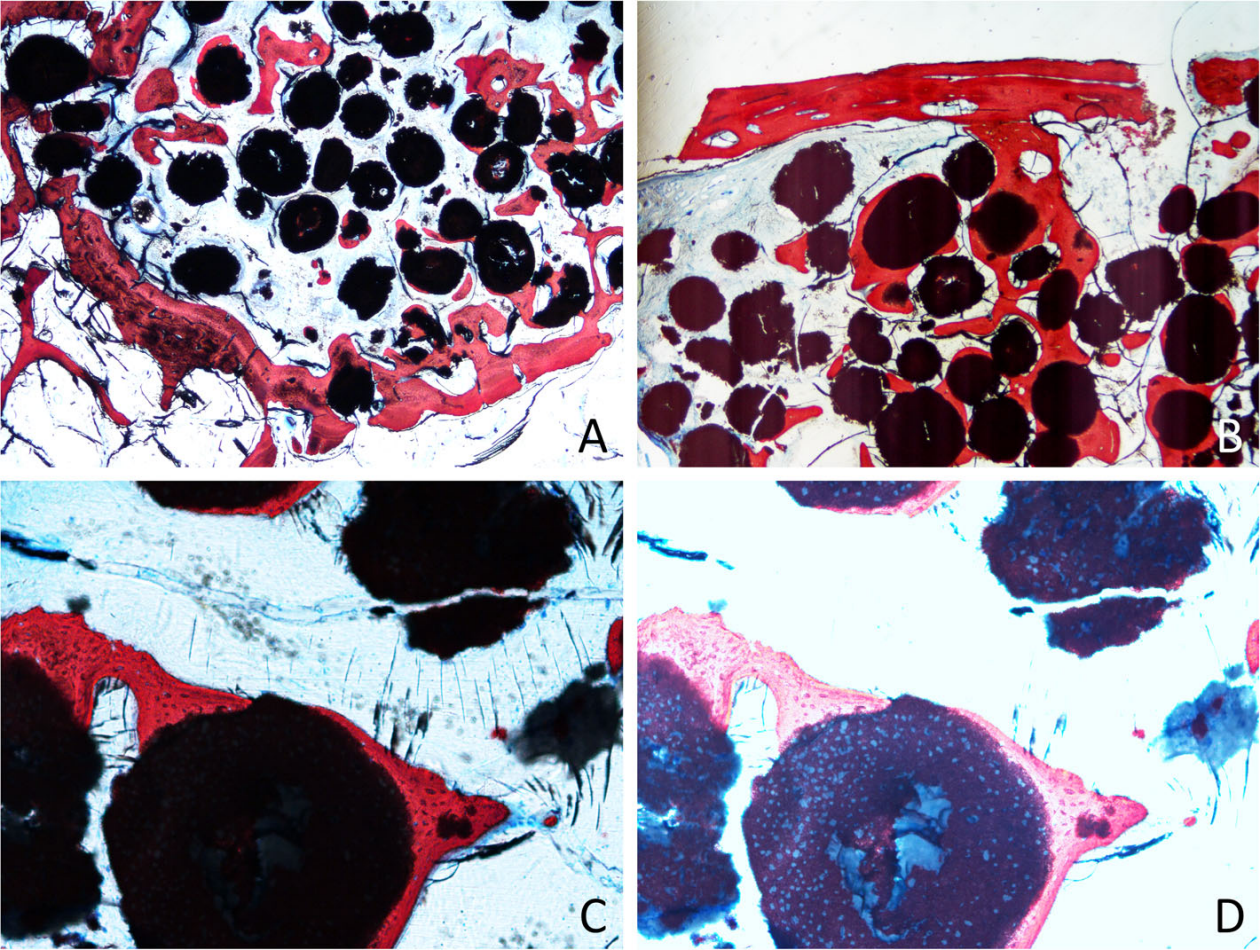
Photomicrographs of ground sections after 4 months of healing. **a** Bone formed from the base of the sinus. **b** Bone plate connected by bridges of the new bone to the close-to-window region. **c** Particle of the graft surrounded by new bone. **d** Overexposed image to show the new bone ingrowth within the granules of biomaterial

The newly formed bone was found surrounding and partially interpenetrating the particles of the biomaterial and in continuity with the lateral and medial walls of the sinus (Fig. 4a) and, when present, in continuity with the repositioned bony window (Fig. 4b).

**Fig. 5:**
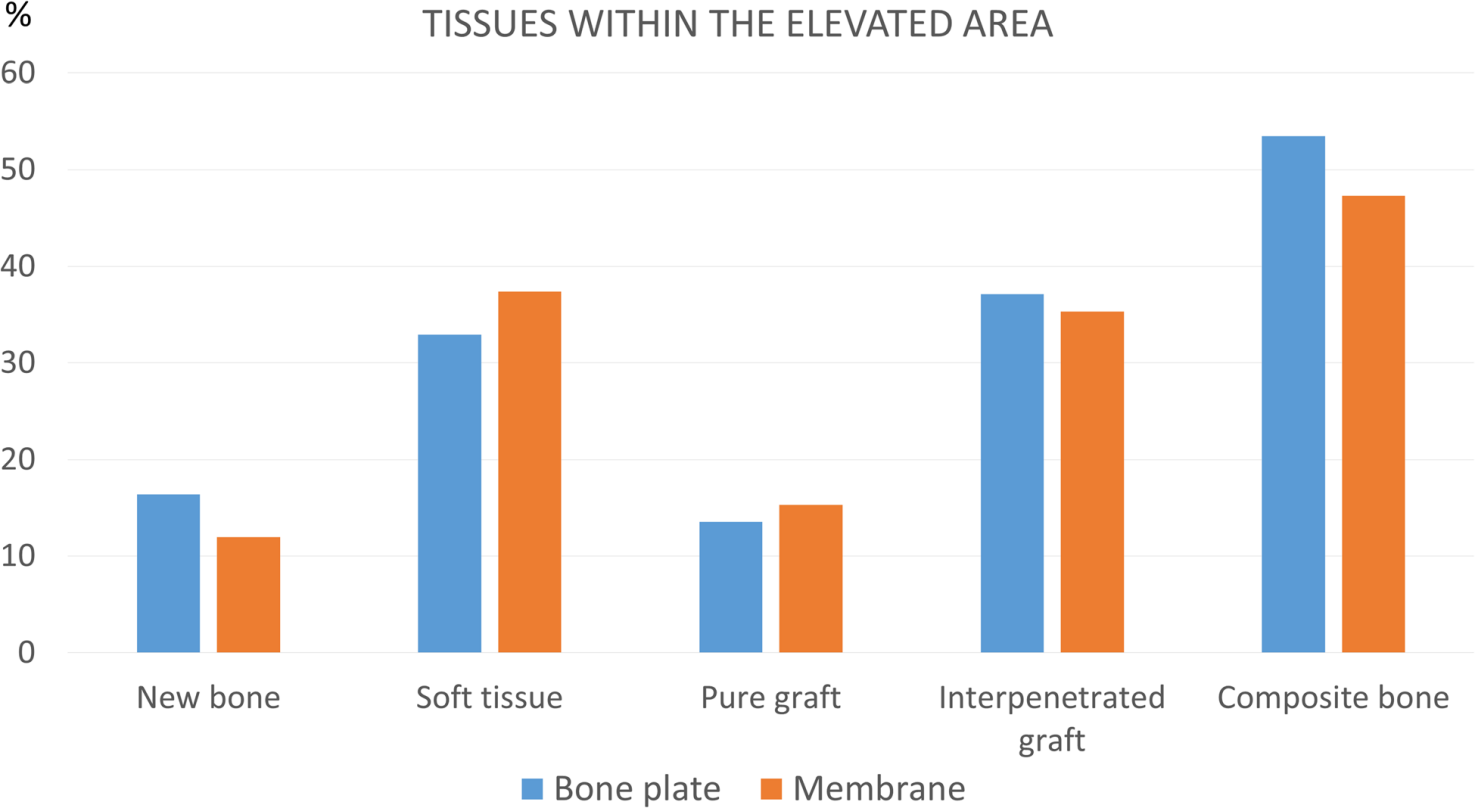
Graph representing the tissue percentages within the elevated area. No statistically significant differences were found

**Fig. 6:**
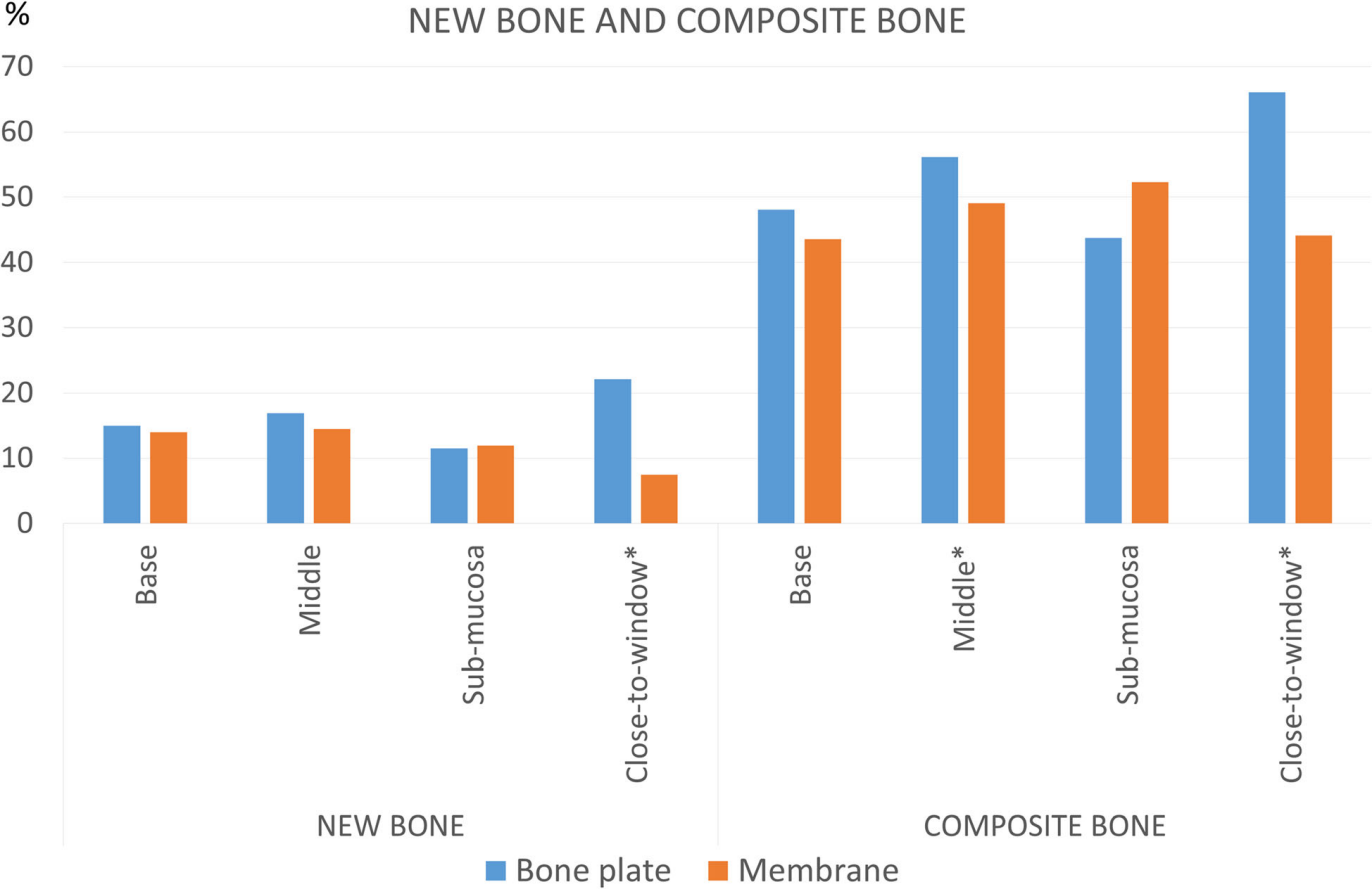
Graph representing new bone and composite bone percentages within the elevated area

After 4 months of healing, the newly formed bone proportions within the augmented sinus were 16.4 ± 5.6% at the test sites and 12.0 ± 3.7% at the control sites. In both groups, large amounts of interpenetrated graft were observed, in which bone propagated within the porosities of the biomaterial at fractions of 37.1 ± 7.5% and 35.3 ± 6.8% at the test and control sites, respectively (Fig. 4c, d). The total bone, composed of newly formed bone and interpenetrated graft, was 53.5 ± 7.6% at the test site (replaced bony window) and 47.3 ± 9.9% at the control site (Fig. 5).

The largest amounts of newly formed bone and total bone (composite bone) were found in the close-to-window region at the test sites. In this region, the proportion of the newly formed bone was 22.1 ± 12.6%* and 7.5 ± 64.5%* (*P* = 0.028) at the test and control sites, respectively. The respective percentages of the total composite bone (new bone plus bone interpenetrated to the graft material) were 66.1 ± 14.7%* at the test sites and 44.2 ± 15.1%* at the control sites (*P* = 0.046). In the middle region, a statistically significant difference in soft tissue amount was seen between the test (29.7 ± 4.9%*) and control (37.5 ± 10.1%*) sites (*P* = 0.046). No substantial differences were found in the submucosal and base regions (Fig. 6).

### Histological analysis in the antrostomy region (Table 2, Fig. 4b)

In the centre of the window area, at the test sites, in the analysed histological sections, the bone plate was still visible in five out of six sheep. It appeared partly remodelled and connected by the new bone formed in the sub-window area. Out of the five bone plates still present, three were bridged to the edges of the antrostomy, while in the two cases no contact was achieved in the observed section. At the control sites, very little bone was found in the centre zone (5.8 ± 2.1%*), while soft tissue was the most represented (52.0 ± 11.8%*, *P* = 0.028). At the test sites, the high content of bone was represented by the bony window (61.5 ± 46.9%).

At the edge of the antrostomy, a higher content of the new bone was found at the test sites (37.2 ± 37%) compared with the control sites (9.2 ± 9.6%).

In the analysed histological slides, no remnants of cyanoacrylates were observed nor indirect signs of inflammatory response. All the percentages of various tissues in the antrostomy area can be seen in Table 2.

**Table 2:** Percentages of various tissues in the antrostomy area after 4 months of healing. Mean values ± standard deviations (*P* values) and median (25%; 75% percentiles)

		Bone	Soft tissue	Pure graft	Interpenetrated graft	Composite bone
**Replaced window**	**Center**	61.5 ± 46.9 82.5 (22.2; 96.9)	21.7 ± 22.6* 17.5 (3.1; 34.4)	2.3 ± 4.3* 0.0 (0.0; 2.3)	7.9 ± 19.3 0.0 (0.0; 0.0)	69.3 ± 38.5 82.5 (57.6; 96.9)
	**Edge**	37.2 ± 37.0 21.6 (16.8; 55.8)	41.0 ± 39.8 33.6 (9.2; 64.9)	5.3 ± 7.3 1.6 (0.0; 9.1)	13.8 ± 19.0* 6.7 (0.0; 19.7)	54.8 ± 34.1 62.9 (37.5; 69.7)
**Membrane**	**Center**	5.8 ± 2.1 (*P* = 0.116) 5.0 (4.2; 7.1)	52.0 ± 11.8* (*P* = 0.028) 51.5 (42.5; 60.5)	12.7 ± 10.7* (*P* = 0.043) 13.8 (4.6; 17.2)	29.4 ± 8.3 (*P* = 0.075) 30.3 (26.4; 33.5)	35.3 ± 10.0 (*P* = 0.075) 35.3 (30.6; 41.8)
	**Edge**	9.2 ± 9.6 (*P* = 0.116) 6.5 (5.0; 9.0)	40.7 ± 18.3 (*P* = 0.917) 36.5 (29.6; 53.9)	16.2 ± 12.8 (*P* = 0.116) 15.9 (10.1; 17.9)	34.0 ± 16.3* (*P* = 0.046) 31.7 (20.0; 45.8)	43.2 ± 16.8 (*P* = 0.600) 44.3 (30.2; 53.0)

*P < 0.05

## Discussion

The aim of the present study was to compare the healing of the augmented sinus in large animals, where the antrostomy was covered by a polylactic membrane or a repositioned bone plate, both secured with cyanoacrylate.

No statistically significant differences were found between test and control sites in the bone formation within the augmented space. This outcome is in agreement with a similar study in rabbits, in which sinus augmentation was performed bilaterally using a collagenated cortical cancellous bone [17]. In that study, the antrostomy at the control site was covered with a collagen membrane, while at the test site it was closed with the repositioned bony window. The healing was evaluated after 2, 4, and 8 weeks, and no differences were found at any of the periods assessed.

In the present study, different regions within the elevated area were evaluated. A statistically significant difference was found in the close-to-window region where the new bone was found at higher proportions at the repositioned sites compared with the polylactic membrane sites. This outcome could have limited clinical relevance since implants are positioned in the middle of the augmented area. In the study mentioned above [17], no differences were found in any of the regions examined, including in the close-to-window region. However, remaining defects within the antrostomy were seen at the collagen membrane sites. Similarly, in the present study, a high content of connective tissue was found in the antrostomy in the polylactic membrane group, while the repositioned bone plate protected the antrostomy from soft tissue ingrowth.

The results from the present study are not in complete agreement with those from the other experiments [16, 22, 23]. In an experiment in rabbits [22], sinus augmentation was performed bilaterally. At one site, the elevated volume was filled with DBBM and the antrostomy was covered with a collagen membrane. The other sinus was filled with a clot and the antrostomy closed, with the repositioned bony window secured with a screw. The healing was evaluated after 1, 2, 4, 6, and 8 weeks, and a higher content of the new bone was found at the repositioned sites compared with the collagen membrane sites. No evaluations of specific regions within the sinus were reported. However, it must be considered that no xenograft was placed at the repositioned bone window, so comparisons with the present study may not be suitable. However, in another experiment in rabbits [16], a DBBM graft similar to that used in the present study was placed to augment both sinuses. The antrostomies were covered with either the repositioned bone window or a collagen membrane, and healing was evaluated after 1, 2, 4, and 8 weeks. Statistically significant higher amounts of the new bone were found at the repositioned bone window groups compared with the polylactic membrane groups. In the present study, however, the only statistically significant difference was seen in the close-to-window region. Moreover, the bone window was found connected with bridges to the new bone formed from the central regions and to the edges of the antrostomy, in agreement with the abovementioned experiments [16, 17, 22].

In another similar experiment in sheep, the sinuses were augmented using a similar biphasic calcium phosphate (60% HA, 40% β-TCP) [24], the biomaterial used in the present study. The perforation of the sinus mucosa was performed at the test sites and a collagen membrane was placed to protect the perforation, while at control sites the elevated mucosa was left unprotected. After 12 weeks of healing, the new bone was 16.7% at the test sites and 13.7% at the control sites. These results are similar to those from the present study, which found a proportion of 12–16% of the new bone. In our study, we also found the newly formed bone interpenetrating the resorbing graft granules. This finding was possible by overexposing the slides during microscopic analysis, and this may have been the reason why it was not recognised in the previously discussed study [24]. A simple overlapping between the bone and the biomaterial could be excluded due to the thinness of the slides and the dimension of granules. This finding increased the amount of bone assessed, considering the total bone as the sum of the new bone alone and the new bone interpenetrating the biomaterial. The amount of bone interpenetrating the graft granules was 37.1% and 35.3% at the test and control sites, respectively, and the amount of the total bone was 53.5% and 47.3% at the test and control sites. This was not in agreement with the previous studies, which did not consider the bone interpenetrated to the graft granules.

The residual amount of biomaterial in the abovementioned study [24] was 36.1% and 30.2% at the test and control sites, respectively. In the present study, a higher content of graft was found, reaching a fraction of about 50% at both sites. Approximately 70–73% of the graft at the test and control sites was interpenetrated within the porosities of the biomaterial by the newly formed bone. This peculiarity had been already described for HA/β-TCP [25, 26], showing the high osteoconductivity properties of the graft used in the present study. The biphasic calcium phosphate (BCP) used in this study consisted of 60% HA and 40% β-TCP; these proportions have been previously suggested as a compromise between the solubility of β-TCP and the mechanical strength of HA [25]. The degradation of β-TCP, being more soluble, increases the porosity of the biomaterial that can be filled by the newly formed bone, while the HA remains in the defect and serves as a scaffold.

In the present study, a lesser amount of bone was registered subjacent to the sinus mucosa compared with the other regions. This is in agreement with another experiment, in which a similar material was used for sinus augmentation in sheep [24]. A similar outcome was also reported in another study [27] that used DBBM xenograft for sinus augmentation in sheep. In that study, a collagen membrane was placed subjacent to the elevated sinus mucosa at the test sites, while no membranes were used at the control sites. Lesser amounts of the new bone were found underneath the sinus mucosa compared with the other regions at both the test and control sites. Moreover, similar amounts of the new bone were observed at both the test and control sites in all regions. This finding allowed the conclusion that the presence of the collagen membrane did not influence the results of healing. These results are in agreement with several other experiments that studied the role of the sinus mucosa in the bone formation [28–30].

In this study, a polylactic membrane was used, while Omori et al. [17] used a collagen membrane. The reason is the greater stiffness on the synthetic membrane compared with the collagen one, that could limit the introflection of the membrane in the augmented area. We found no difference in the bone formation, confirming Omori’s results; membrane’s material does not seem to influence bone formation.

In the analysed histological slides, no cyanoacrylate was observed. This outcome is not in complete agreement with the previously mentioned study [17] where few remnants of cyanoacrylate were observed in the gap between the bone plate and the edges of the antrostomy, which seemed to interfere with bone formation, although the adhesive showed a progressive degradation during the analysed periods of healing. The authors suggest the use of a cyanoacrylate with high viscosity. A way to increase the viscosity could be to keep the adhesive in cold conditions, as in our experiment. In the present study, no inflammatory response to cyanoacrylate was seen, confirming what has already been observed in other studies that excluded the cytotoxicity of cyanoacrylate [18] and negative systemic effects [31].

## Conclusion

In conclusion, the repositioning of the bone window after sinus floor elevation in sheep, compared with the use of a resorbable membrane, improved the closure of the antrostomy and led to a greater amount of the newly formed bone in the close-to-window zone of the grafted area. The bone window appeared partially bonded to the newly formed bone. Bridges of new bone from the edges of the antrostomy reached the repositioned bone window. The newly formed bone was found interpenetrating the graft granules. The cyanoacrylate used to close the antrostomy showed no inflammatory response.

The limitations of the present study were the lower phylogenetic level and thicker sinus mucosa of sheep compared with humans. The small number of animals used, complicated by the reduction of the sample from eight to six, was another limitation of the study.

## Data Availability

All data and materials are available for control and consultation contacting the first author (alexperini@studioschweiger.it).

## Abbreviations

BCP: Biphasic calcium phosphate
DBBM: Deproteinized Bovine Bone Mineral
HA: Hydroxyapatite
INC: Infraorbital nerve canal
SD: Standard deviation
β-TCP: Beta-tricalcium phosphate
